# Enhanced LED Performance by Ion Migration in Multiple Quantum Well Perovskite

**DOI:** 10.1021/acs.jpclett.3c02822

**Published:** 2023-12-15

**Authors:** Shir Yudco, Juan Bisquert, Lioz Etgar

**Affiliations:** † Institute of Chemistry, Casali Center for Applied Chemistry and the Center for Nanoscience and Nanotechnology, The Hebrew University of Jerusalem, Jerusalem 91904, Israel; ‡ Institute of Advanced Materials (INAM), Universitat Jaume I, 12006 Castelló, Spain

## Abstract

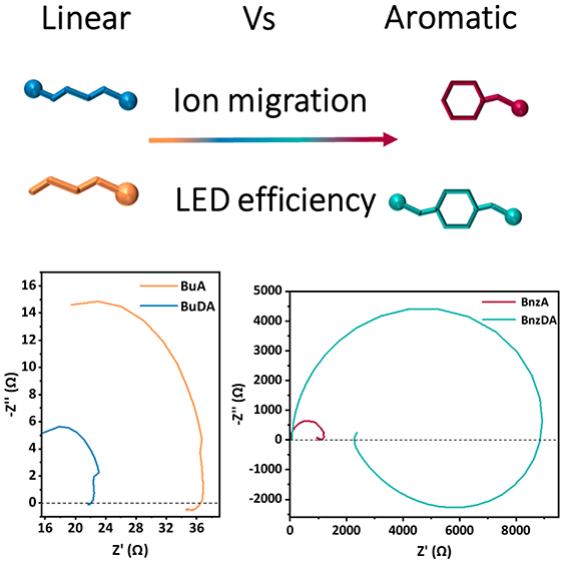

Here we study the effect of ion migration on the performance of perovskite light emitting diodes (PeLEDs). We compared aromatic and linear barrier molecules in Ruddlesden-Popper and Dion-Jacobson two-dimensional perovskites having multiple quantum well (MQW) structures. PeLED devices were fabricated by using the same conditions and architecture, while their electroluminescence properties and ion migration behavior were investigated. Impedance spectroscopy measurements were used to analyze the PeLEDs, which found a direct link between the barrier molecule type, the device efficiency, and ion migration. The best performing LEDs were based on the aromatic barriers, which present dominant inductive impedance, indicating an earlier onset voltage of radiative recombination. These findings present an approach of how to control radiative emission in perovskite LEDs which opens the way for further improvement in PeLEDs and memristors.

Light emitting diodes (LEDs) have become an integral component in today’s lighting industry. LEDs can be found in an enormous variety of applications including lighting,^1,2^ displays,^3^ microscopy,^4^ and sensors^5^ while the commercialization of LEDs has made a major step in reducing energy consumption. In recent years, the LED field has attracted much attention due to the discovery and development of new electroluminescent materials. These new materials aim to reduce manufacturing costs,^6^ improve color purity,^7^ and open new possibilities such as manufacturing flexible devices which show a great promise in organic LED (OLED), light emitting electrochemical cells (LEC), and perovskite based light emitting diodes (PeLEDs).^8,9^ These devices share a similar architecture based on an electroluminescent material layer. Understanding the mechanism of these devices is an important step toward their commercialization.

PeLEDs have risen in recent years as a promising new LED technology with rapidly increasing external quantum efficiency (EQE).^10^ Perovskite has the general structure of AMX_3_, consisting of a monovalent cation at the A site, divalent cation at the M site, and a halide anion at the X site. The ionic nature of the perovskite material presents several opportunities such as simple fabrication processes, tunable band gap, and dimensionality.^11−13^ However, the ionic nature also leads to phenomena such as ion migration, induced by external electric field or photoexcitation.^14,15^ This phenomenon was observed as the hysteresis curve in solar cells at early stages of development^16,17^ which affect the long-term stability. Recently ion migration in perovskite presents the possibility to develop memristors^14,18^ turning the previous disadvantage to a possible new technology. Another beneficial property can arise from the combination of both ionic and electronic conductivity in perovskite. Light emitting electrochemical cell (LEC) operation is based on ionic movement creating an internal PN junction, which has some similarity to the ion migration phenomena in PeLED, therefore it can be assumed that the ion migration in PeLEDs can increase the radiative recombination.

In LEC the bias scan leads ionic charges to transport toward the interface of the emitting layer with the selective contacts, creating a doping of charges at the opposite interface of the emitting layer, with a neutral zone in-between.^19,20^ It was found that the size of the neutral layer can influence exciton quenching, affecting the luminescence intensity of the cells.^21^ PeLEDs and LEC show similar behavior of ion migration, revealed by inductive behavior on impedance spectroscopy (IS) measurements, offering a possible explanation to the PeLED operation mechanism.^22^

In this work, we hypothesize and provide confirmation that the ion migration process in perovskites improves the performance of PeLEDs. Changes in the perovskite composition are known to influence the activation energy of ion migration.^23^ For example, the effect on ion migration of different halides^24^ and cations^25^ was studied, while quasi-two-dimensional (2D) perovskite remains mostly unexplored in this aspect. The quasi-2D structure is formed by a mixture of two cations in the A site, the initial small monovalent cation and a large organic cation (also known as the barrier molecule). The ratio between the two A site cations determines the dimensionality of the perovskite layer. Here we study the connection between the barrier molecule in quasi-2D perovskite, ion migration, and their influence on the performance of PeLED. In order to isolate the effect of the barrier molecule the same device architecture was used, using PEDOT:PSS and MoO_3_ doped F8 as the hole and electron transporting layers respectively as can be seen in Figure 1b (PEDOT:PSS-poly(3,4-ethylenedioxythiophene) polystyrenesulfonate, F8-poly(9,9-di-n-octylfluorenyl-2,7-diyl)). Four different barrier molecules were investigated: butylammoniun (BuA), benzylammonium (BnzA), 1,4-butanediammonium (BuDA), and 1,4-benzenedimethanammonium (BnzDA), (as illustrated in Figure 1a).

**Figure 1:**
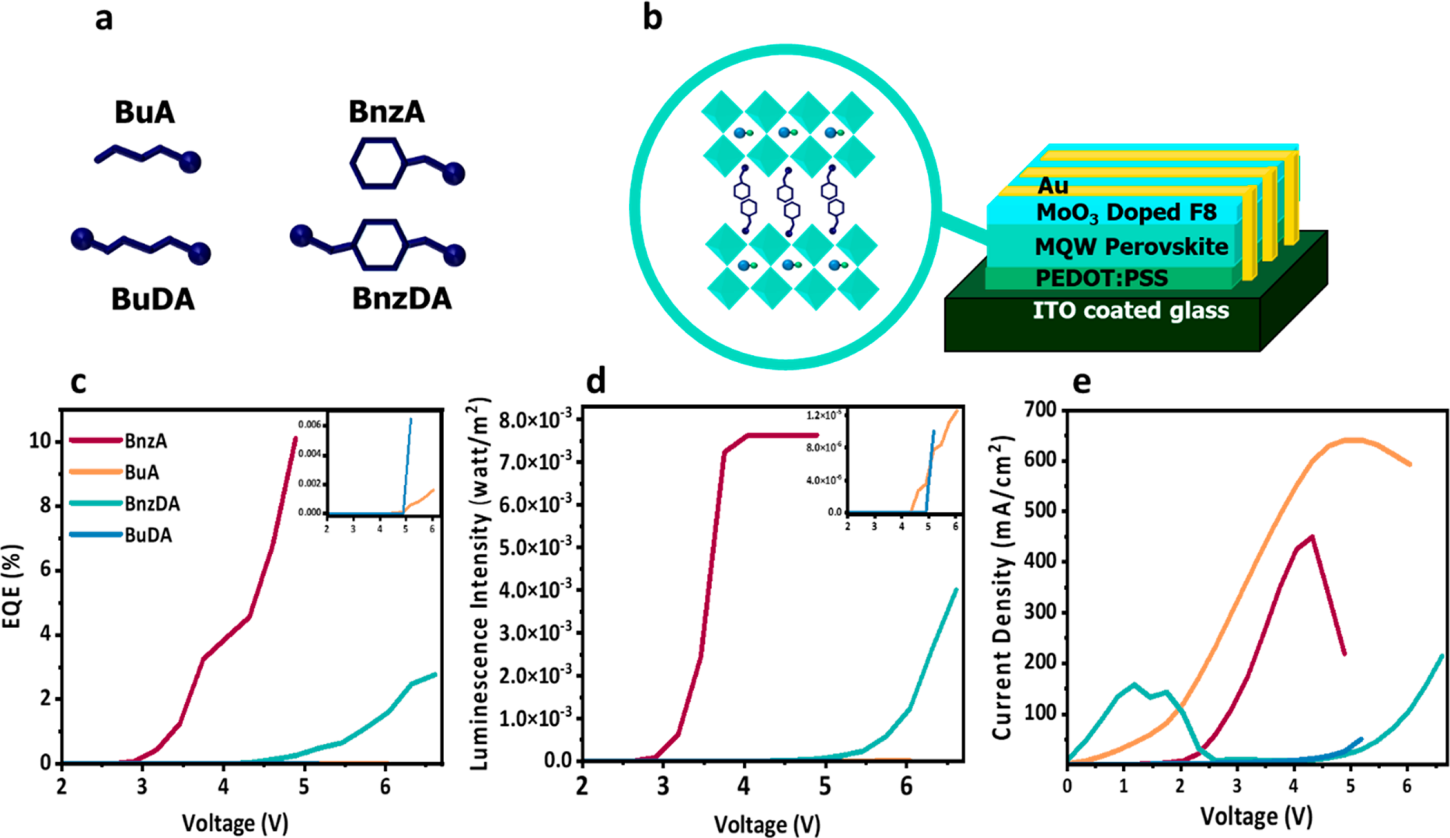
Schematic illustration of (a) the barrier molecules used in this work: butylammoniun (BuA) benzylammonium (BnzA), 1,4-butanediammonium (BuDA), and 4-benzenedimethanammonium (BnzDA). (b) The LED device architecture fabricated in this work. Electroluminescence (EL) measurements of devices with different barrier molecules tracking different device parameters vs applied voltage. (c) External quantum efficiency (EQE) of the aromatic and linear barriers (inset). (d) EL intensity measured in watt/m^2^ of the aromatic and linear barriers (inset) and (e) current density in mA/cm^2^.

The performance of the LED devices based on the barrier molecules shows different results. LED devices using aromatic barriers performed better than those using linear barriers. The external quantum efficiency (EQE) of BnzA and BnzDA is higher by 3 orders of magnitude compared to the linear barrier molecules, achieving 10% and 5%, respectively, while BuA and BuDA demonstrate EQE of 0.002% and 0.006%, respectively (Figure 1c). The most significant improvement can be observed in the luminescence intensity in Figure 1d, indicating enhanced radiative recombination in the case of the aromatic barriers. However, the current density in Figure 1e shows different current behaviors affecting LED performance. Figure 2 compares the device’s current density and electroluminescence performance under a bias scan. Evidence of leakage current can be observed for all of the barrier molecules by the increase in the current density prior to the radiative recombination, which is represented by the luminescence intensity. In the aromatic barriers a second process of nonradiative recombination can be observed (Figure 2a,c) by the decrease in the current density prior to the turn-on voltage of the device. This nonradiative current is more dominant in BnzDA, as seen by the sharp decrease in the current density, providing an explanation for the difference in efficiency between the aromatic barriers.

**Figure 2:**
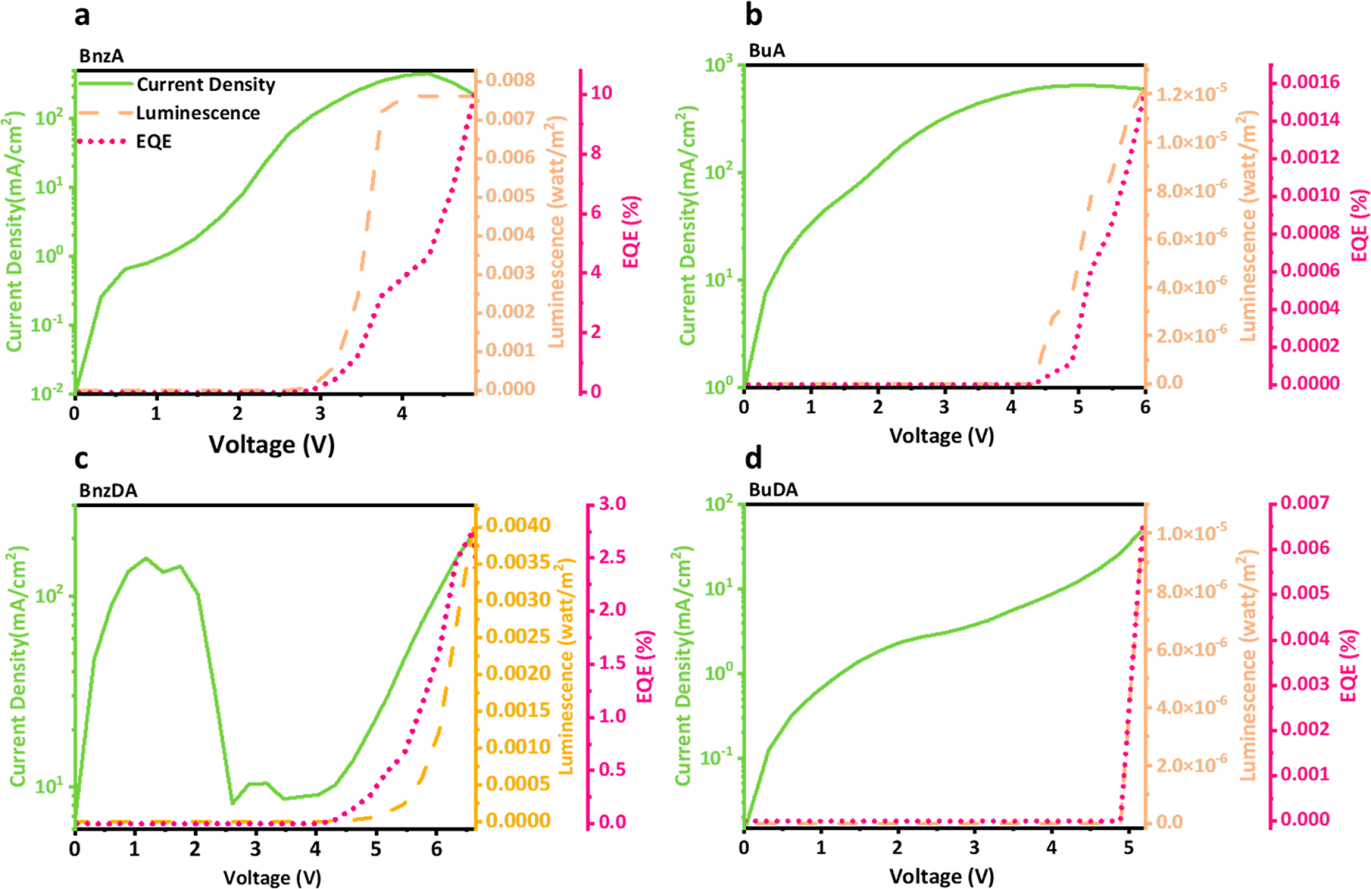
Measurements of current density, EL intensity, and EQE vs voltage for devices using the different barrier molecules: (a) BnzA, (b) BuA, (c) BnzDA, and (d) BuDA.

In order to understand the electrical current mechanism inside the devices, impedance spectroscopy (IS) measurements were performed for all the barrier molecules. The measurements were conducted in the dark and at a voltage range of 0.1 V–2 V, using 0.1 V steps. The orthonormal Nyquist plots for all the barriers can be seen in Figure 3a-d. As voltage is increased and recombination increases, the decrease of the size of the arcs is observed due to a decrease of the recombination resistance. We remark that in our experiments reliable impedance features are only viable up to 2 V. Thereafter the resistance becomes small and the spectra are noisy. The current voltage-curves are observed to display additional features at larger voltages, including a decrease and later increase associated with the radiative recombination, as mentioned before (Figure 2).

**Figure 3:**
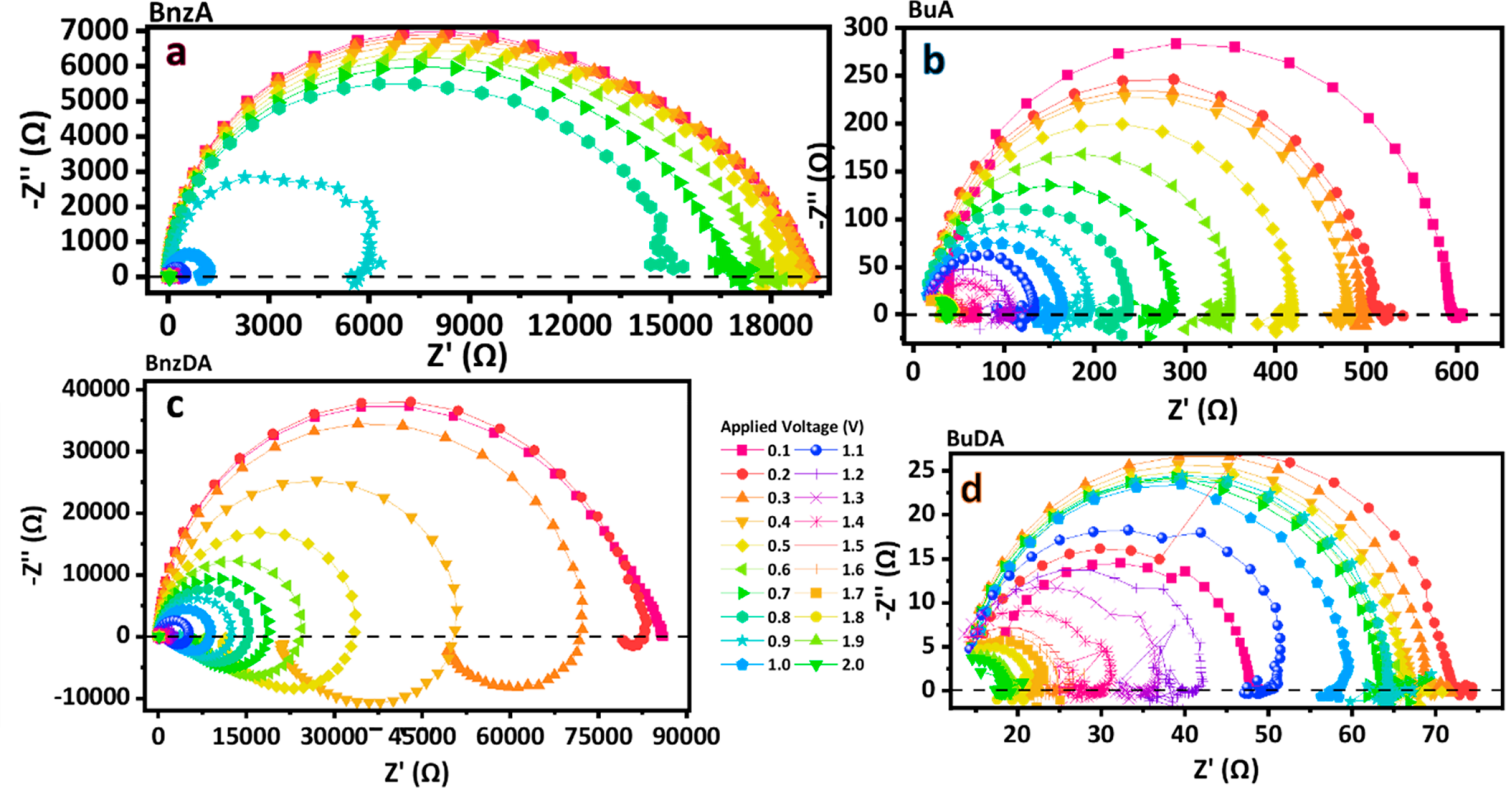
Impedance spectroscopy (IS) measurements. Orthonormal Nyquist plots for all barrier molecules: (a) BnzA (b) BuA, (c) BnzDA, and (d) BuDA.

In the case of the aromatic barriers, a steep decrease in the total resistance can be observed at 0.8 V for the BnzA (Figure S1a) and at 0.2–0.5 V (Figure S1c) for the BnzDA. This steep decrease, which indicates the onset of the radiative recombination process responsible for the higher EQE, is absent in the nonaromatic barriers.

To obtain a better understanding of the different features of light emission, we analyze the evolution of the impedance spectra. An important phenomenon observed is the transition of the impedance behavior depending on the voltage regime, that has been reported previously in halide perovskites.^26^ At low voltage measurements, a single arc can be seen at all frequencies with the standard parallel structure *RC* in terms of the equivalent circuit. As the voltage is increasing, we can recognize another feature at low frequencies: an arc in the fourth quadrant. This feature represents the appearance of inductive behavior. The change can be seen gradually through the measurement as observed in Figure 4, starting with fully capacitive behavior. With a further increase in the voltage, a small negative capacitance feature starts to appear indicating a transformation stage, until a full induction arc appears in the fourth quadrant. The corresponding frequency ranges can be observed in the capacitance Bode plots in Figure S2.

**Figure 4:**
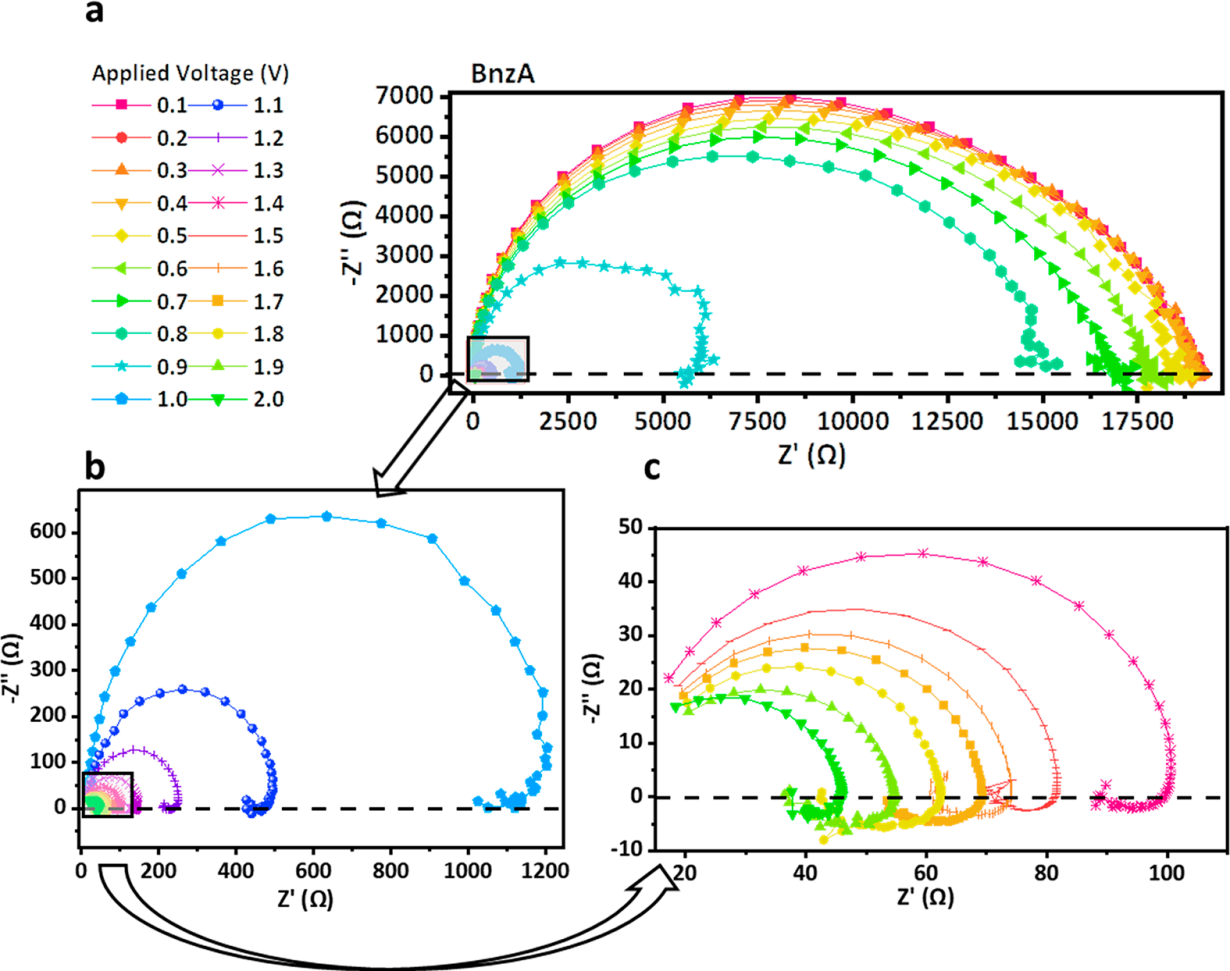
Orthonormal Nyquist plots for BnzA IS measurements with enlargement at different voltage ranges: (a) Full measurement range from 0.1 to 2.0 V. (b) 1.0 V to 2 V. (c) 1.4 V to 2.0 V.

In general the inductive behavior is caused by the combination of fast and slow current modes.^27^ This feature is equivalent to the famous “negative capacitance” often observed in halides perovskites^28,29^ associated with ion migration effects phenomena^30,31^ related to the ionic nature of the perovskite. More generally the negative capacitance is found in many solution-processed optoelectronic devices.^30^ In halide perovskites the appearance of the inductive feature is explained by the presence of surface polarization that influences recombination at some onset voltage.^32,33^

Remarkably, the onset of inductive behavior occurs in a different way for the different compositions, based on the barrier molecule type and correlated to the device performance, as indicated in Table 1. In BnzA all three stages, fully capacitive, transformative, and inductive, can be observed (Figure 4) while in BnzDA the transition into a full fourth quadrant arc occurs at very low voltage, almost immediately after the voltage is applied. However, in the linear barriers, the full fourth quadrant arc does not appear; only the transformation stage can be seen in the measurement, without the transfer to a complete inductive behavior as in the case of the aromatic barriers. We remark that the linear barriers show larger grain size (Figure S3) than the aromatic barriers which can lead to a reduction of halide anion migration due to fewer grain boundaries.^23,34^ The larger number of small grains in the aromatic barriers appears to be highly beneficial for the surface polarization phenomena that boost the radiative recombination effect. This is in stark contrast to halide perovskite solar cells, where the decrease of resistance associated with the inductor harms the fill factor.^35^

**Table 1:** Comparison of the Onset of Light Emission and Inductor Behavior for All Barrier Molecules

barrier molecule	onset of inductor voltage (V)	onset of light emission (V)
BnzA	1.0	2.9
BuA	0.9	4.6
BnzDA	0.2	4.3
BuDA	1.0	5.1

To rationalize the interplay of the inductive process and the radiative recombination current, we present for the first time an electro-optical model for a PeLED that permits an interpretation of the observed behaviors. The model is inspired in recent papers that describe the chemical inductor feature in halide perovskites^36−39^ and iontronic conducting channels.^40,41^

A schematic representation of the model is presented in Figure 5a. The total current *j*_tot_ as a function of voltage *u* is composed of a leakage nonradiative current *j*_*L*_ = *g*_*L*_*u* with conductance *g*_*L*_, a radiative current *j*_rad_, and a capacitive current with capacitance *C*_*g*_
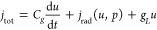
1

**Figure 5:**
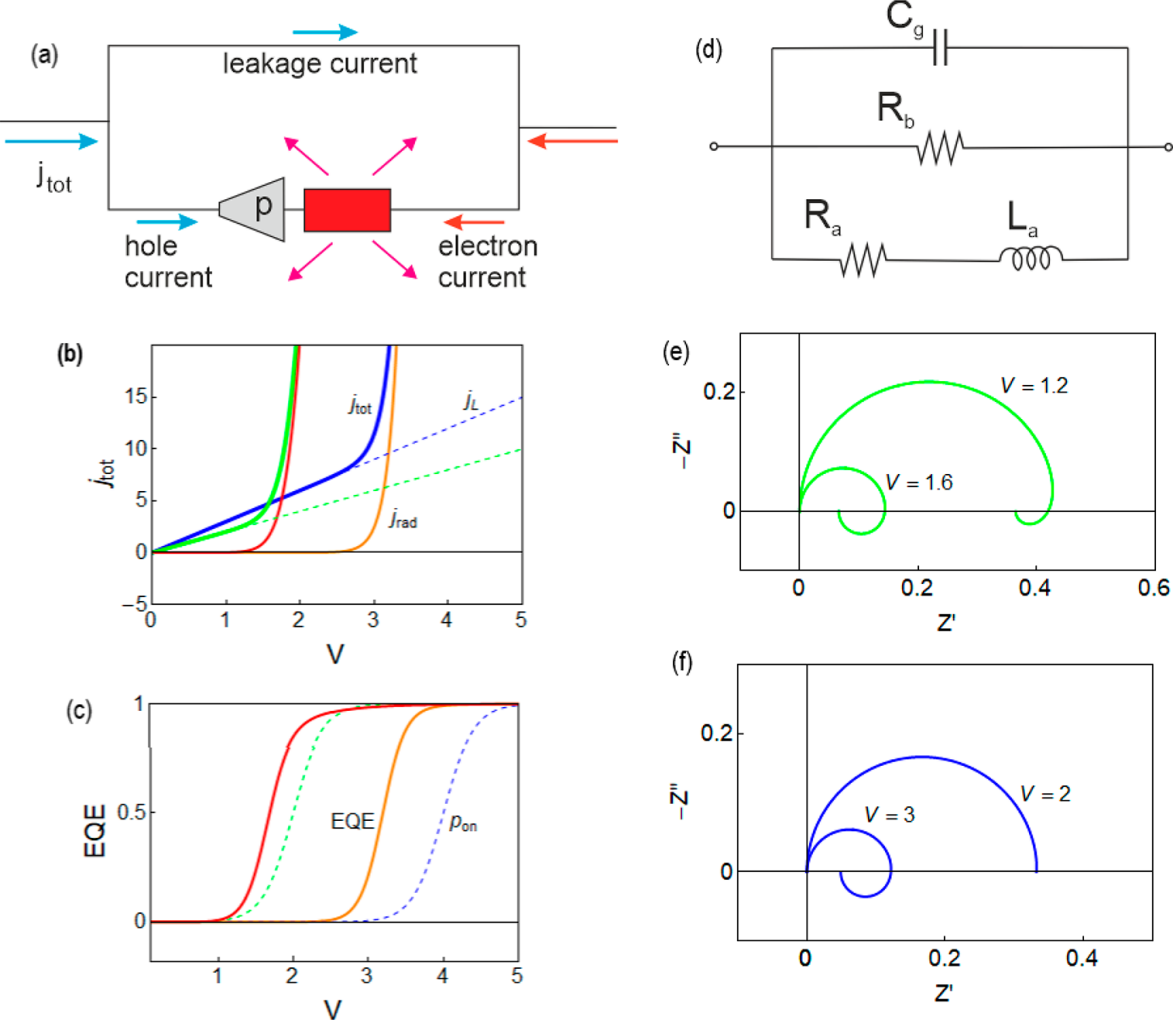
(a) Scheme of the model. The total current is divided in two parallel pathways, a radiativeless current of a single carrier through the device, and a pathway of electron–hole radiative recombination. The second pathway is controlled by an activation barrier described by a gating variable *p* that turns on at some voltage *V*_on_ with a delay time τ_*k*_ related to the ionic accumulation process. (b) Radiative, leakage (dashed), and total current. (c) EQE and *p*_on_ (dashed). (d) Equivalent circuit model. (e-f) Evolution of impedance spectra at different applied voltages. Parameters *g*_rad_ = 1; *U*_0_ = 0.5; *V*_*r*_ = 0.6; *V*_*p*_ = 0.2. Green-red (b-c-e): *V*_on_ = 2.0; *g*_*L*_ = 2. Blue-orange (b-c-f): *V*_on_ = 4.0; *g*_*L*_ = 3.

The radiative current is the product of a conductance *g*_*rad*_, a drift term (*u*-*U*_0_) with built-in voltage *U*_0_, and an exponential recombination term with voltage rise parameter *V*_r_. Additionally *j*_rad_ is activated by the gating variable *p*.
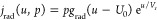
2

This expression with a conductivity term, a drift term, and an onset variable *p* is a standard feature of ion channel models.^40,41^

The temporal dynamics of the variable *p* is delayed by the need of the ionic diffusion effect to activate the recombination process. This is described by the equation with characteristic time τ_*k*_
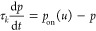
3This last equation produces the chemical inductor effect.^26,40,41^ When the variable *p* comes to equilibrium with the applied voltage *u* it has the expression
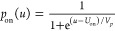
4Therefore, the *p* variable passes from 0 to 1, and therefore blocks the radiative current when the voltage is *u* < *U*_on_. *V*_*p*_ is the steepness of the rise of *p*_on_.

Figure 5b shows the form of the total current in two cases (green and blue) and the radiative recombination current (red and orange). At low voltage, the nonradiative leakage current dominates the total current, but at the voltage *V*_on_ the intrinsic barrier is overcome, as shown in Figure 5c, and the light emission current occurs. In the second case shown in Figure 5b (blue), the voltage *V*_on_ is 2 V larger; hence, the radiative recombination is delayed to a larger voltage. This example shows the comparison of the behavior of samples with aromatic and linear barriers, as the former have a consistent lower onset voltage than the latter.

To analyze the IS results, we calculate the impedance function of the model of eqs 1–4 using standard methods. The impedance *Z* at the angular frequency ω can be written quite generally in terms of the variable *s* = *iω* as^41,42^
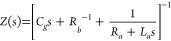
5We have introduced two resistances, *R*_*a*_ and *R*_*b*_ and an inductor *L*_*a*_, defined as
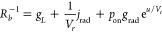
6
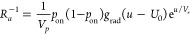
7
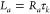
8

The equivalent circuit of eq 5 is shown in Figure 5d and corresponds to the general structure of a chemical inductor.^27^ The evolution of the impedance spectra for the two cases of Figure 5b is shown in Figure 5e,f. We remark on the onset of the inductor at the voltage *V*_on_. Clearly in the case with larger *V*_on_, the inductor is delayed in the voltage scale, with respect to the case where the barrier is overcome at a lower voltage. Therefore, we can assume that in the aromatic barriers, the LED has a lower turn on voltage *V*_on_ than in the linear barriers, leading to earlier light emission.

The question is the meaning of the onset variable *p* that activates the recombination current at the voltage *V*_on_. According to the model of Figure 5a the sample contains two parallel morphologies. One is a phase of direct transport of one carrier from one electrode to the other. The other pathways are formed by barriers in the multiple quantum well structure. Each quantum well requires a delayed polarization influenced by ionic diffusion to permit the entrance of electrons and holes that enable radiative recombination and light emission. This phenomenon is well-known in LECs where the initial ion migration enhances the recombination zone.^21,22^ The extent of polarization with respect to voltage is described by the function *p*_on_, and the ion diffusion controlled time of formation of the recombination-activated state is τ_*k*_.

According to the measurements of current, luminescence, and IS, the linear barriers require a higher activation voltage than the aromatic barriers, as already mentioned. Another feature observed in the aromatic cases of BnzDA and BnzA is a decrease of the resistance before the onset of radiative recombination. This behavior is explained in Figure S4 of the SI. This is a typical feature of biological and artificial ionic channels,^40,41^ and occurs when the built-in voltage that controls carrier drift in the electrical field has a similar value of the turn-on voltage of the gating variable *p*.

Finally a puzzling behavior when comparing the results of BnzA and BnzDA, is that the former has a lower onset of radiative recombination, but the latter has a lower onset of the inductor. We explain this result in Figures S5 and S6, on the basis of a different radiative recombination parameter *g*_rad_. That is, BnzDA has a lower *V*_on_, which makes the inductor appear very early, but it also has a weak recombination efficiency, which causes the light emission to occur later.

The differences between BnzA and BnzDA can also be observed in the device stability (Figure S7). The efficiency of the devices was tracked during consecutive measurements, turning the device on and off while tracking the changes of the EQE. It can be seen that BnzA maintained over 70% of its original efficiency, while BnzDA efficiency increased initially and then decreased to less than 1%. Figure S8 shows that the capacitance response of all the devices is similar and has the form of a nearly constant capacitance; hence, the main differences are related to the inductive effect and radiative recombination onset. The lower stability observed in BnzDA can be related to the intrinsic poor recombination efficiency, involving a larger ionic reorganization, as compared to BnzA. Nevertheless, despite the degradation, the impedance measurement appears reproducible and reliable regarding the shape of spectra, as can be seen in the measurements in Figure S9.

This work presents the influence of the perovskite composition having different barrier molecules on PeLED performance. The IS measurement of the various barrier molecules shows an inductive behavior, which indicates an ion migration process. In the case of aromatic barriers, ion migration was pronounced, according to the appearance of a large inductive arc, which results in higher EQE than the nonaromatic barriers. Similarly to LECs, and in contrast to solar cells, an extent of ion migration is beneficial to enhance the radiative recombination process that is desired in a LED. However, excessive ion migration can affect the device stability.^43^ This work shows the importance of understanding the functionality of the barrier molecules in a PeLED toward the development of a new generation of LEDs.

## Experimental Methods

### Precursor Synthesis

Butylammoniun (BuA), benzylammonium (BnzA), 1,4-butanediammonium (BuDA), and 1,4-benzenedimethanammonium (BnzDA), were synthesized from commercial precursor using butylamine (Aldrich 99.5%), benzylamine (Aldrich 99%), 1,4-diaminobutan (Acros 99%), and p-xylylenediamine (Aldrich 99%), respectively. The precursor was dissolved in absolute ethanol (Holland Moran 99.8%) with a slow drip of hydrobromic acid (48 wt % in water, Aldrich) added in excess to create the barrier salt. All barriers were cleaned by three washes in diethyl ether (Bio-Lab) and recrystallized in ethanol absolute.

### Device Fabrication

Glass substrates with conductive indium tin oxide (ITO) (15Ω, Automatic Research) were cleaned in four sonication baths of Hellmanex (2%), deionized water, acetone, and ethanol followed by oxygen plasma treatment for 15 min. The substrates were coated with poly(3,4-ethylenedioxythiophene) polystyrenesulfonate (PEDOT:PSS) (Al 4083 Ossila) using dynamic spin coating at 6000 rpm for 30 s and annealed at 140 °C for 15 min. The PEDOT:PSS layer was treated with a spin of ethanol absolute and deionized water solution in a 4:1 ratio, at 4000 rpm for 40 s and annealed at 120 °C for 5 min.

Perovskite solutions were prepared in a nitrogen-filled glovebox. The precursor stoichiometric ratio was determined by the stoichiometric ratio of the Ruddlesden–Popper structure (R-NH_3_)_2_(A)_*n*-1_M_*n*_X_3*n*+1_ or the Dion-Jacobson structure (NH_3_-R-NH_3_)(A)_*n*-1_M_*n*_X_3*n*+1._ Using the synthesized barrier, MABr (GreatCell Solar Company) and PbBr_2_ (Aldrich ≥98%). The solutions were dissolved in dimethyl sulfoxide (DMSO, Aldrich 99.7% extra dry) and dimethylformamide (DMF, Aldrich, anhydrous 99.8%). The solvent ratios and concentrations were determined based on device optimizations for each barrier. Using 0.8 M in 2:3 DMSO:DMF ratio for BuA, 1 M in only DMSO for BnzA, 1 M in 4:1 DMSO:DMF ratio for BuDA, and 1.2 M in 1:4 DMSO DMF ratio for BnzDA, heated overnight at 60 °C.

The perovskite was spin coated at 3000 rpm for 30 s for BnzA, BuA, and BuDA, and 1000 rpm for 10 s followed by 5000 rpm for 60 s for BnzDA. The devices were annealed for 20 min at 100 °C. Poly(9,9-di-n-octylfluorenyl-2,7-diyl) (F8, Aldrich) was dissolved in 1 mg to 75 μL of chlorobenzene (CB, Aldrich 99.8% extra dry), and spin-coated at 3000 rpm for 30 s, then MoO_3_ was thermally evaporated in vacuum of ∼10–7 Torr for 125 s followed by thermal evaporation of 70 nm Au metal contact.

*Electroluminescence (EL)*. The measurements are a combination of two simultaneous measurements, emission measurements and current–voltage (I–V) measurements. I–V curve measurements were performed using a Keithley model 2400 digital source meter. EL measurements are performed using an F1000-VISNIR optic fiber with cosine receptor, StellarNet BLACK-Comet spectrometer with CRX-100 partially depleted absorber photodetector.

*Scanning Electron Microscopy (SEM)*. The measurements were performed using Magellan Extra High-Resolution SEM using a FEI (field emission instruments), The Netherlands. The measurement conditions were 5 kV.

*Impedance spectroscopy (IS)*. The measurements were performed using an Autolab Potentiostat-Galvenostat (PGSTAT) with a FRA32 M LED driver. A Nova 1.1 software program was used to collect and analyze the obtained data. The IS measurements were conducted at different bias voltages with perturbation of 10 mV from 1 MHz to 0.1 Hz, under dark conditions.

## Supporting Information Available

Total resistance vs applied DC voltage for all barrier molecules. Top view images (SEM) of the perovskite layer surface, using the different barrier molecules. Additional information on the decrease of the resistance and earlier onset of the inductor in the sample with larger onset of luminescence. Stability measurements for PeLEDs using the aromatic barrier molecules. Capacitance vs applied DC voltage for all barrier molecules. Bode plots of capacitance vs frequency for BnzA IS measurements. Stability measurement tracking the changes to the IS spectra due to EL measurement of the device (PDF)
