# Supplementary material for: Enhanced LED Performance by Ion Migration in Multiple Quantum Well Perovskite
Source: J Phys Chem Lett. 2023 Dec 15;14(51):11610–7. doi: 10.1021/acs.jpclett.3c02822 (PMC11163466; doi:10.1021/acs.jpclett.3c02822)
Supplement: Supplementary file 1 — jz3c02822_si_001.pdf [file jz3c02822_si_001.pdf]

Enhanced LED Performance by Ion Migration in Multiple Quantum Well Perovskite

Shir Yudco[1], Juan Bisquert[2] and Lioz Etgar[1,].*

[1] Institute of Chemistry, Casali Center for Applied Chemistry and the Center for Nanoscience and Nanotechnology, The Hebrew University of Jerusalem, Jerusalem 91904, Israel

[2] Institute of Advanced Materials (INAM), Universitat Jaume I, 12006 Castelló, Spain

* lioz.etgar@mail.huji.ac.il

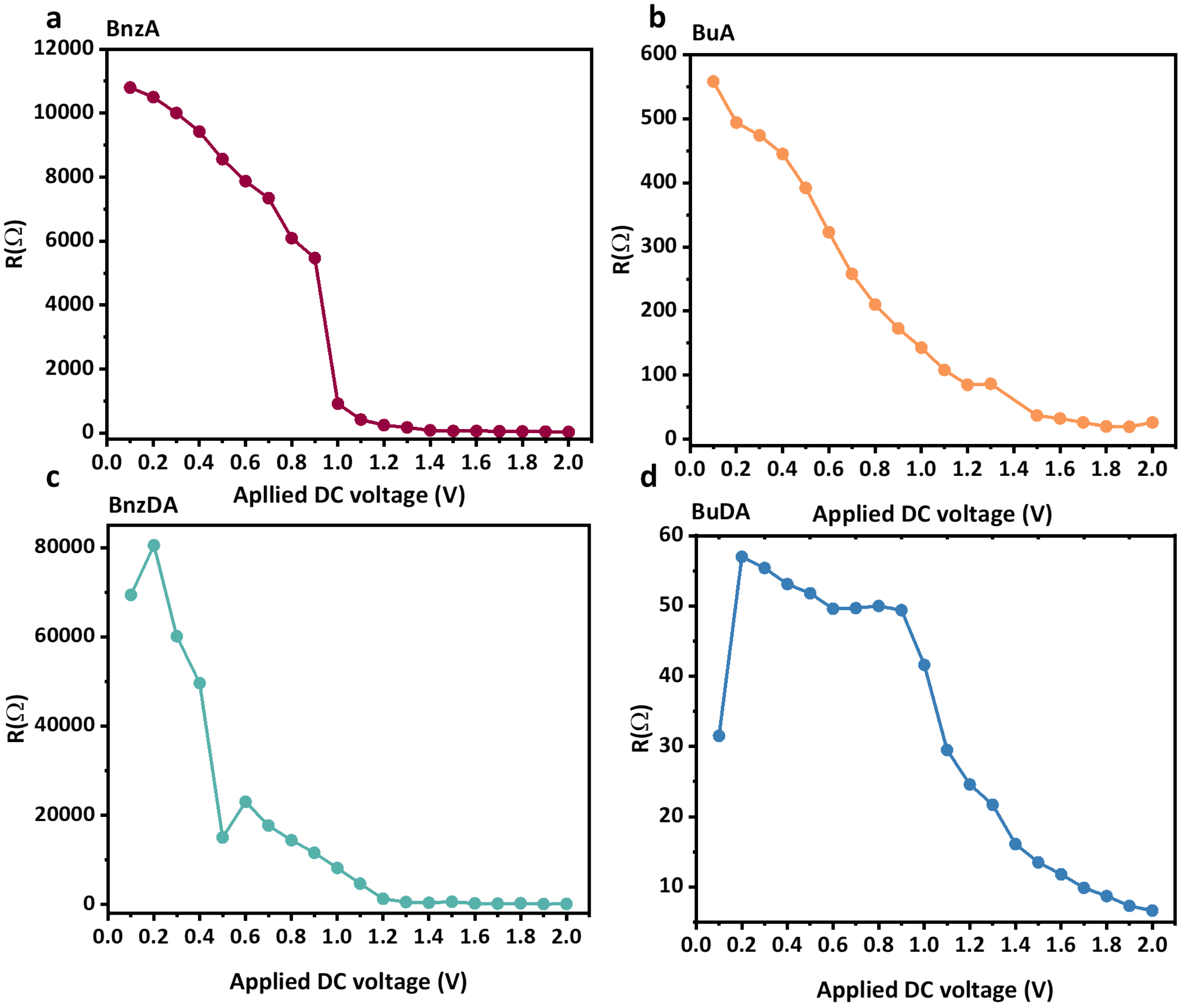

Figure S1. Total resistance vs applied DC voltage for all barrier molecules: (a) BnzA (b) BuA, (c) BnzDA and (d) BuDA.

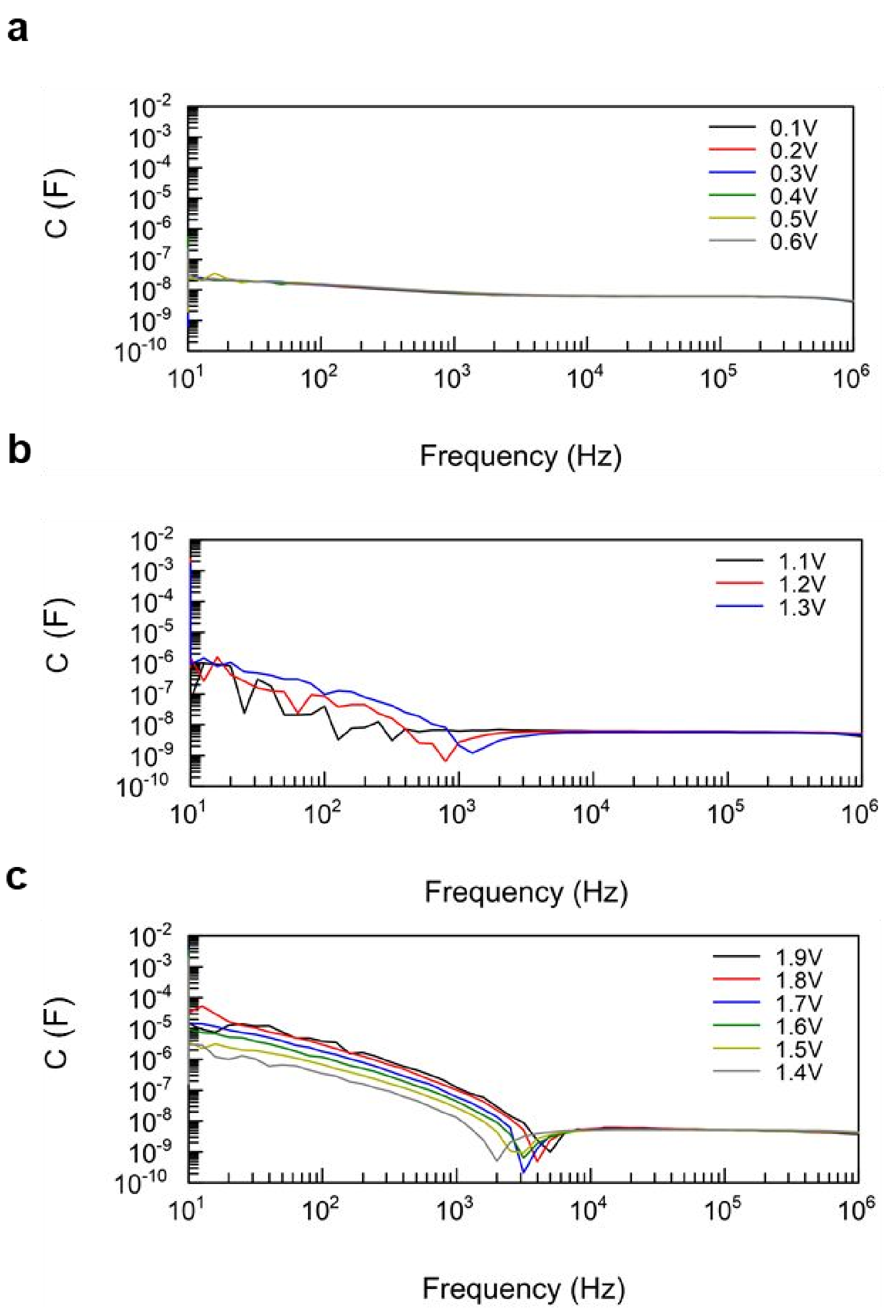

Figure S2. Bode plots of capacitance vs Frequency for BnzA IS measurements at different voltage ranges from (a) 0.1V to 0.6V (b) 1.1V to 1.3 and (c) 1.4V to 1.9V, showing the appearance of inductive behavior with a gradual change as the voltage is increased.

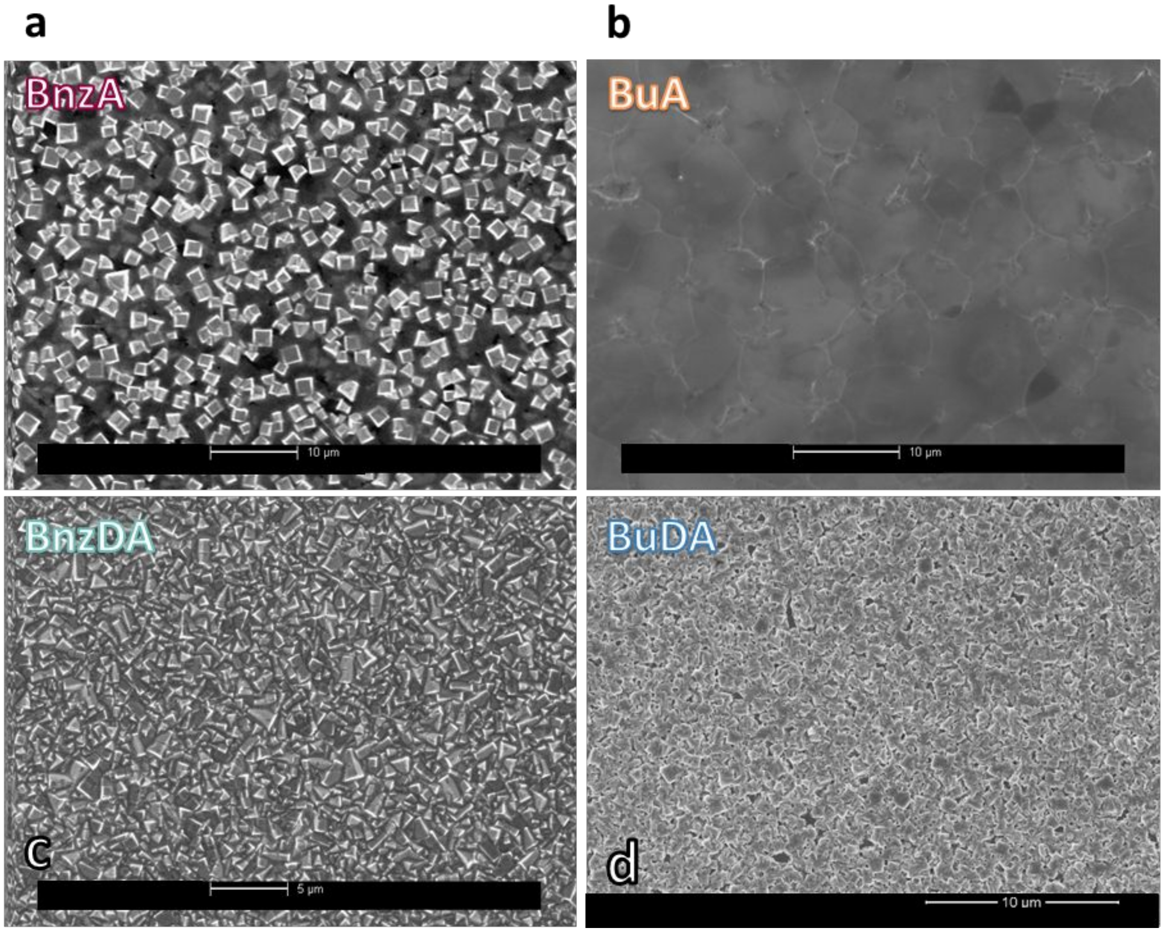

Figure S3. Scanning Electron Microscopy (SEM) top view images of the perovskite layer surface, using the different barrier molecules (a) BnzA, b) BuA ,(c) BnzDA, and (d) BuDA.

Decrease of the resistance

Figure S4 shows a regime of decline of the resistance at larger applied voltage as observed experimentally in the aromatic samples. This feature occurs if the turn on voltage is similar to the built-in voltage, $V_{on} \approx U_0$.

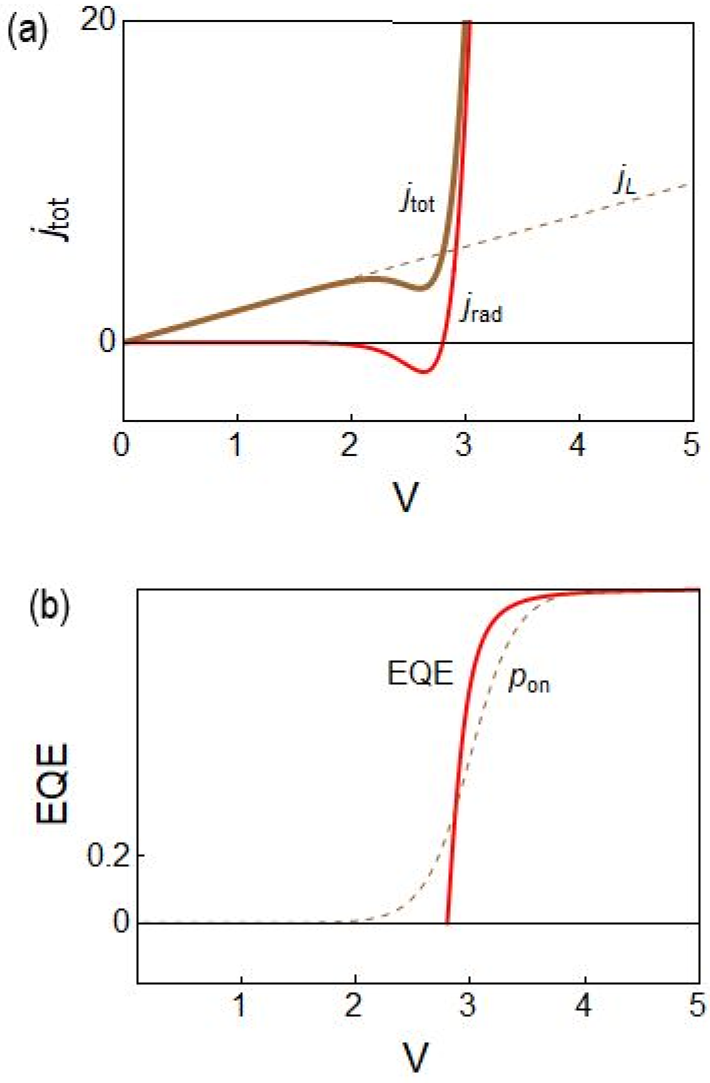

Figure S4. (a) Radiative, leakage (dashed), and total current. (b) EQE and p_{on} (dashed). Parameters $g_{rad} = 1; V_r = 0.6; V_p = 0.2$; $V_{\text{on}} = 3.0; \text{U}_0 = 2.8$; $g_L = 2$.

Earlier onset of the inductor in the sample with larger onset of luminescence.

Figs. S5 and S6 show this feature that is observed experimentally in the aromatic samples. If the recombination rate parameter g_{rad} is similar, then the onset of the inductor is correlated with the onset of EL, as discussed in the figure 5 of the main text. However, if g_{rad} becomes small due to less intrinsic radiative recombination, then the EL will be delayed with respect to the inductor.

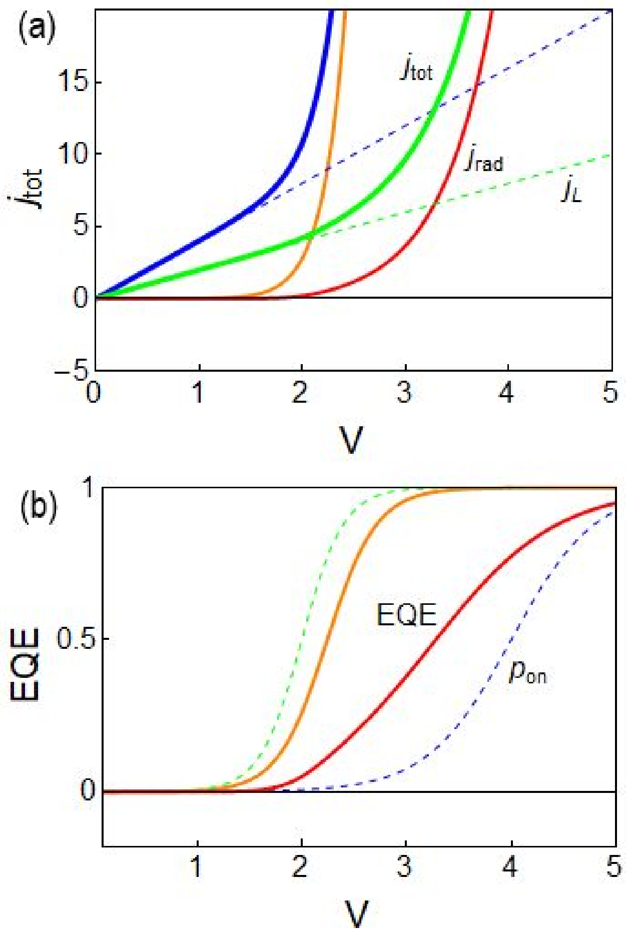

Figure S5. (a) Radiative, leakage (dashed), and total current. (b) EQE and p_{on} (dashed). Parameters $U_0 = 0.5$; $V_r = 0.6$;$V_p = 0.2$. Green-red: $g_{rad} = 0.01$;$V_{\rm on} = 2.0$;$g_L = 2$. Blue-orange: $g_{rad} = 10$; $V_{\rm on} = 4.0$;$g_L = 4$.

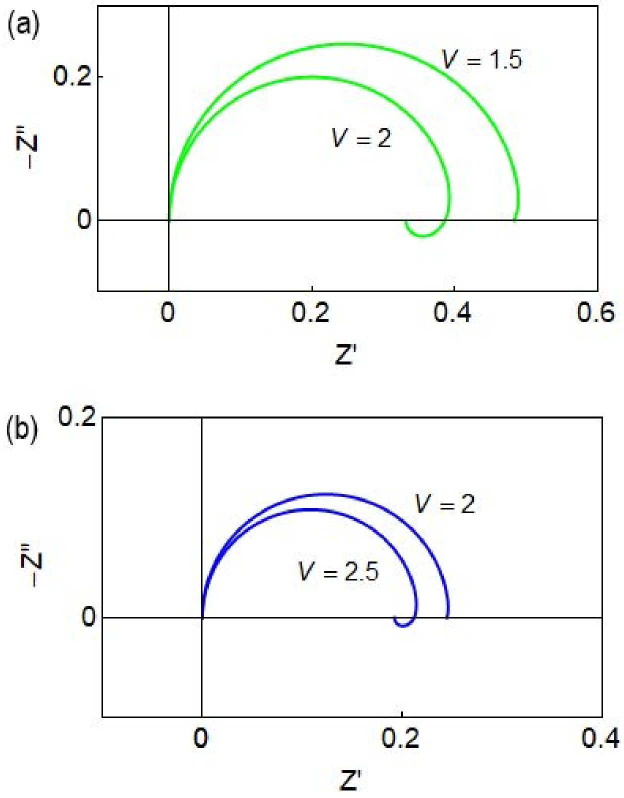

Figure S6. Evolution of impedance spectra at different applied voltages. Parameters;$U_0 = 0.5$; $V_r = 0.6$;$V_p = 0.2$. (a) $g_{rad} = 0.01$; $V_{\text{on}} = 2.0$;$g_L = 2$. (b) $g_{rad} = 10$; $V_{\text{on}} = 4.0$;$g_L = 4$.

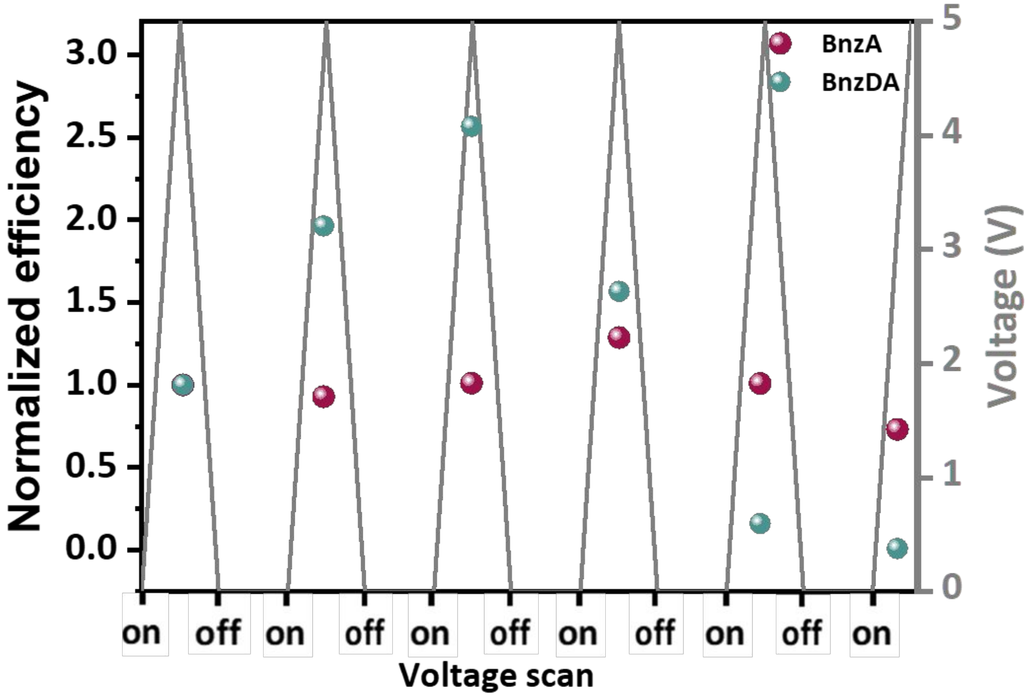

Figure S7. Stability measurements for PeLEDs using the aromatic barrier molecules under consecutive electroluminescence measurements, turning the PeLED on and off using cycles of 0Vto 5V.

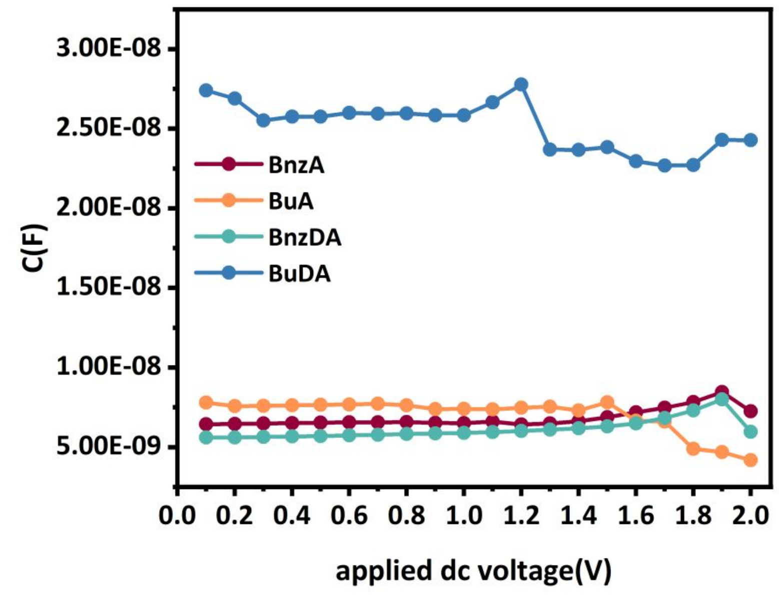

Figure S8. Capacitance vs applied DC voltage for all barrier molecules.

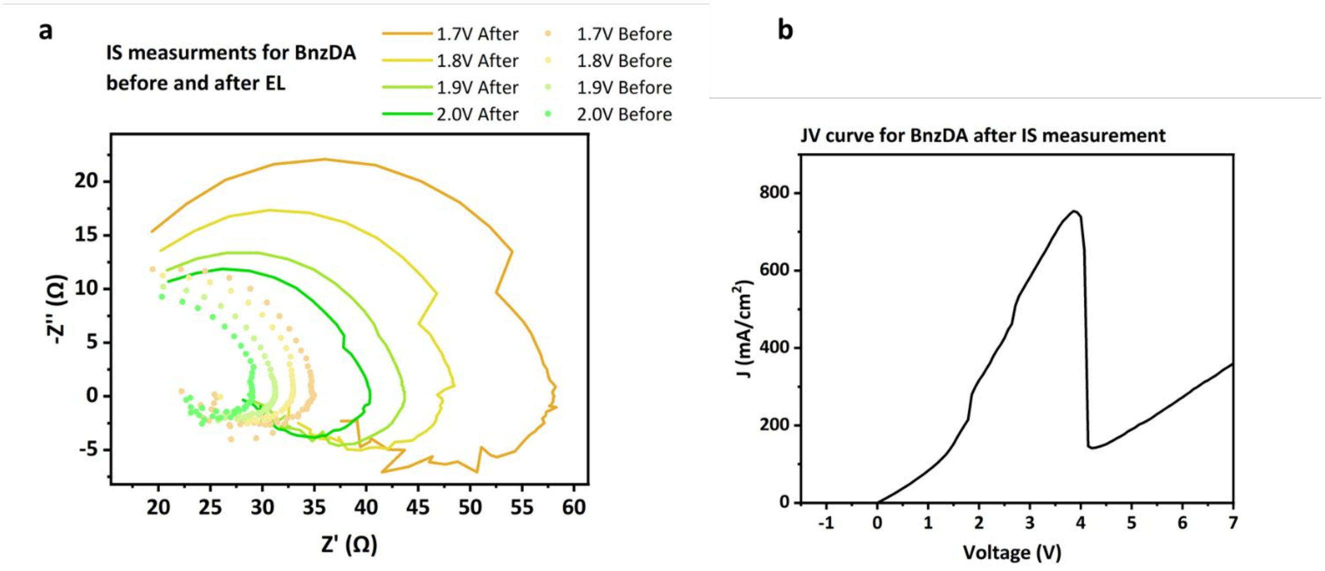

Figure S9. Stability measurement trucking the changes to the IS spectra due to EL measurement of the device (a) Orthonormal Nyquist plots for BnzDA IS measurements before and after EL measurement. (b) JV curve of the EL measurement preformed after IS measurement.
